# Machine Learning Models for Indoor Positioning Using Bluetooth RSSI and Video Data: A Case Study

**DOI:** 10.3390/s25216640

**Published:** 2025-10-29

**Authors:** Tomás Mamede, Nuno Silva, Eduardo R. B. Marques, Luís M. B. Lopes

**Affiliations:** 1 Department of Computer Science, Faculty of Sciences, University of Porto, Rua do Campo Alegre 1055, 4169-007 Porto, Portugallmlopes@fc.up.pt (L.M.B.L.); 2 INESC TEC, Campus da Faculdade de Engenharia da Universidade do Porto, Rua Dr. Roberto Frias, 4200-465 Porto, Portugal

**Keywords:** indoor positioning system, machine learning, ensemble learning, multimodal data

## Abstract

Indoor Positioning Systems (IPSs) are essential for applications requiring accurate location awareness in indoor environments. However, achieving high precision remains challenging due to signal interference and environmental variability. This study proposes a multimodal IPS that integrates Bluetooth Received Signal Strength Indicator (RSSI) measurements and video imagery using machine learning (ML) and ensemble learning techniques. The system was implemented and deployed in the Hall of Biodiversity at the Natural History and Science Museum of the University of Porto. The venue presented significant deployment issues, namely restrictions on beacon placement and lighting conditions. We trained independent ML models on RSSI and video datasets, and combined them through ensemble learning methods. The experimental results from test scenarios, which included simulated visitor trajectories, showed that ensemble models consistently outperformed the RSSI-based and video-based models. These findings demonstrate that the use of multimodal data can significantly improve IPS accuracy despite constraints such as multipath interference, low lighting, and limited beacon infrastructure. Overall, they highlight the potential of multimodal data for deployments in complex indoor environments.

## 1. Introduction

Indoor Positioning Systems (IPSs) aim to determine the positions of people or objects inside buildings. They have numerous applications in the management and safety of residential, commercial, and industrial infrastructures. Current IPS implementations use a variety of technologies, including Inertial Measurement Units (IMUs), Radio–Frequency Identification (RFID), Near-Field Communication (NFC), Wi-Fi, Bluetooth, UWB, Visible Light Communication (VLC), and cameras, complemented by algorithms that estimate positions from the hardware-generated data [1,2]. Recently, there has been considerable interest in using multimodal data and machine learning (ML) techniques to process sensor data and produce models that serve as the core components of an IPS [3,4,5].

**Figure 1:**
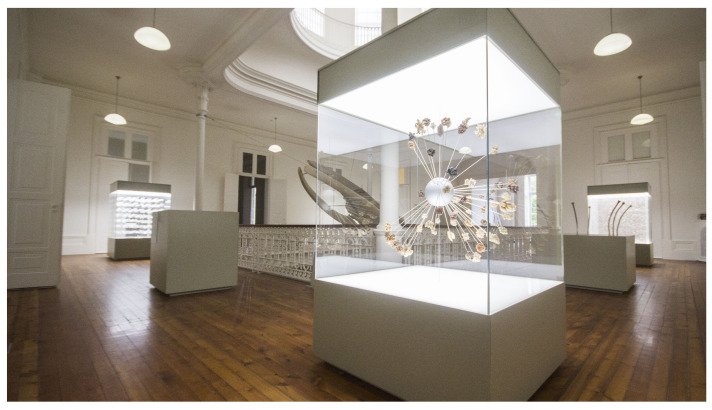
Panoramic view of the atrium at the Hall of Biodiversity. Credits: Ecsite/Ciência Viva/MHNC-UP. Courtesy of the Natural History and Science Museum of the University of Porto.

Here, we present a proof of concept for an Indoor Location-Based System (ILBS) developed for the Hall of Biodiversity (hereafter, the Hall), shown in Figure 1, part of the Natural History and Science Museum of the University of Porto (https://mhnc.up.pt/galeria-da-biodiversidade/, accessed on 18 July 2025). The system is designed to accurately predict visitors’ locations and suggest additional experiences such as videos, augmented reality, games, or targeted content for the museum store. ILBS aims to predict the region of interest (ROI) where a visitor is located, a room in the museum, or a subsection of a room, using RSSI signals from Bluetooth beacons and video feeds, with both types of data captured using smartphones as users roam the museum space. The building where the Hall is located is an architectural landmark of the city. Therefore, the IPS that supported the ILBS was designed to be non-intrusive, easy to deploy, and low-cost. The setup includes a mesh of Bluetooth beacons deployed in the rooms on the first floor of the building. These are managed through a software platform that processes beacon telemetry and enables bidirectional communication. This was an evolution of previous work, in which we utilized the Hall and its gardens—the Botanical Garden of the University of Porto—as a test bench [6,7,8].

In this setting, we used a prototype mobile application to register the Received Signal Strength Indicator (RSSI) data from the Bluetooth beacons. The application also recorded video data, from which frames were later extracted. The RSSI and video data were annotated with the ground truth ROI labels, the required granularity for the ILBS. The data were collected by visiting the premises and walking around the rooms in predefined patterns to acquire sufficient RSSI and image data for each ROI. The raw data were stored on the device’s local disk and later transferred to cloud storage for further processing and construction of the datasets.

We extend preliminary work [9] by using the scikit-learn toolkit to derive several ML models (AdaBoost, Decision Tree, Gradient Boost, k-Nearest Neighbors, Linear SVM, Multi-layer Perceptron, Random Forest, and Radial Basis Function) for the RSSI dataset. For video data, we employ transfer learning to derive deep learning models from the image dataset, based on state-of-the-art pre-trained TensorFlow CNN architectures. Finally, we combined the best RSSI and CNN models using ensemble techniques.

This paper presents the complete process—from beacon deployment and data collection to dataset construction, model derivation, and evaluation. The main contributions of this paper can thus be summarized as follows:

1. The RSSI and video datasets;
2. ML models based on RSSI data;
3. ML models based on video data and using transfer learning;
4. Hybrid ML models combining RSSI and video models using ensembles;
5. A comprehensive evaluation of all models;
6. The datasets and Python notebooks used in the analysis [10];
7. A demonstration that the use of multimodal data improves the accuracy of positioning models in contexts where control of the environment and deployment strategies are significantly constrained.

The remainder of the paper is structured as follows. Section 2 describes the current state of the art in IPS, focusing on ML techniques. Section 3 details our materials and methods concerning the deployment of the Bluetooth beacon mesh at the Hall, the construction of the RSSI and video datasets, and finally the generation of the models from the datasets using scikit-learn and TensorFlow. Section 4 presents the results obtained with the ML models for the RSSI, video, and hybrid models. Finally, Section 5 discusses the main results and suggests some directions for future research.

## 2. Related Work

The field of Indoor Positioning Systems (IPSs) has experienced significant advancements over the past 20 years, driven by the increasing demand for accurate indoor navigation solutions across various areas [1,2]. This progress is the result of technological developments at both the hardware and software levels. Most early systems were based on Radio–Frequency Identification (RFID) for tracking resources in indoor environments. Inertial Measurement Units (IMUs), typically employed in the context of Dead–Reckoning methods, were also used. Later systems progressively used existing Wi-Fi infrastructures to determine location by employing RSSI and trilateration techniques. Other systems relied on application-specific infrastructures, such as Bluetooth Low Energy (BLE) and Ultra-Wideband (UWB), to improve accuracy. Some of these IPSs use Time-of-Flight (ToF) techniques to achieve centimeter-level accuracy [11]. A key milestone in the field was the realization that multimodal sensor fusion techniques, which combine data from different sensors (e.g., Bluetooth RSSI and video from CCTV or smartphones) [12,13], can further refine accuracy. The introduction of video as an extra input then led to the application of existing computer vision techniques to IPS. Another major research milestone was demonstrating the positive impact of machine learning techniques (e.g., k-Nearest Neighbors (kNN), Random Forests, neural networks) and, more recently, deep learning, on the development of accurate IPSs [3,4,14]. In this context, machine learning is combined with existing hardware technologies such as Wi-Fi [15,16,17,18,19], Bluetooth [20,21], UWB [22,23], Visible Light Communication (VLC) [24], video [25,26], or multimodal combinations of these. Lately, machine learning techniques such as attention-based mechanisms and multimodal learning have also been shown to be effective for use in IPSs [27,28,29,30].

Closer to our case study, the literature provides examples of IPSs designed for museums. Koniusz et al. [31] present an artwork identification system based on a CNN-derived model. The model was trained with a dataset composed of images of different art pieces. Each piece identified by the model implicitly provides the application with the user’s position. Majd and Shafabakhsh [32] demonstrate how ML-derived indoor positions can positively impact visitor experience, e.g., by providing automatic guide methods. Girolami, La Rosa, and Barsocchi [33] built a dataset based on RSSI collected during 32 museum visits of 10 artworks with different smartphones and visiting paths. The same authors [34] present two proximity detection algorithms calibrated using data crowd-sourced from the mobile phones of museum visitors. The devices gather RSSI readings from Bluetooth tags and relay them to a back-end server, where the data is used to calibrate the algorithms. The authors note the clear improvement in positioning accuracy using this crowd-sourcing architecture. Ferrat et al. [35] create a dataset spanning 90 objects distributed over 13 rooms in a museum. The dataset is based on RSSI measurements with no multimodal data. Associated with the dataset, they also provide proximity- and kNN-based models for position prediction.

While there are several important contributions to developing IPSs for real-world applications in the literature, many assume freedom to control environmental variables (e.g., lighting) and to deploy infrastructure at will (e.g., Bluetooth beacons). This is hardly the case with many museum installations, such as the one we present. Given these constraints, we demonstrate that using multimodal data, such as that gathered from visitors’ ubiquitous mobile phones, can help improve the accuracy of positioning models. Moreover, as can be verified from recent work [33,35], the field lacks datasets based on real-world scenarios that can be used to develop and test positioning algorithms and IPSs. This work contributes to this effort by building and providing a new multimodal dataset based on the Hall of Biodiversity, along with all associated processing scripts.

## 3. Materials and Methods

We now describe the deployment of the Bluetooth beacon mesh at the Hall, the construction of the RSSI and video datasets, and finally the generation of the models from the datasets using scikit-learn and TensorFlow.

RSSI (Received Signal Strength Indicator) is a measure of the quality of the received radio signal by a device. RSSI measurements are obtained by radio transceivers whenever they scan the medium for other devices. In the context of Bluetooth, RSSI values are measured in decibels (dBm) on a logarithmic scale. Values typically range from 0 (strong signal) down to a protocol-defined minimum (weak or undetectable signal). The RSSI for a device is affected not only by the distance to the receiver but also, and—most importantly—by the phenomenon of multipath propagation; that is, the possibility that a radio signal may reach the receiver by following two or more paths. Inside buildings, radio signal propagation is influenced by walls and objects. These reflect, refract, or diffract radio waves in varying amounts, distorting signals and enabling multipath propagation. Signals following different paths can interfere destructively, negatively impacting RSSI.

### 3.1. Beacon Deployment

The first step towards building the RSSI and video frame datasets involved planning and deploying the Bluetooth beacon infrastructure. The following prerequisites guided the choice of hardware:

- Using low-cost, off-the-shelf devices;
- Using well-established protocols such as Eddystone or iBeacon;
- Compatibility with most mobile devices;
- Low maintenance;
- Long battery life.

**Figure 2:**
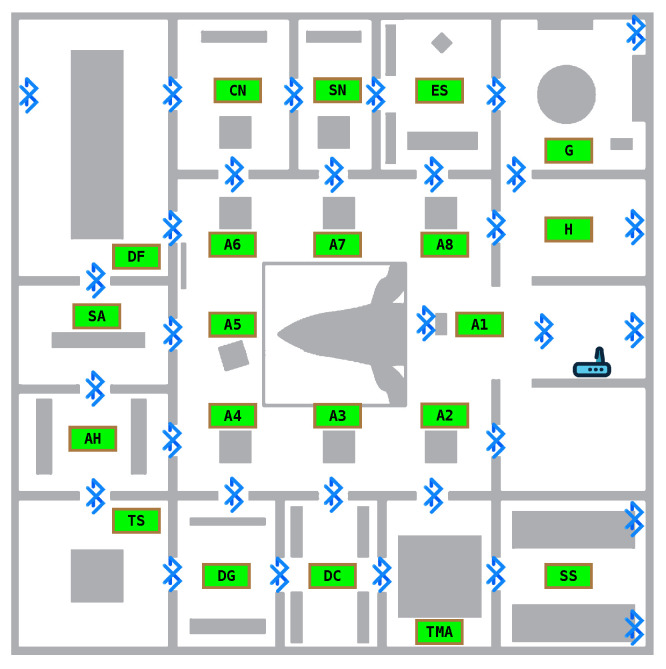
Beacon deployment and ROIs on the first floor of the Hall. Each ROI has a theme and is named accordingly. Their names are as follows: A3—What’s that smell?; A6—Ethical Principle; A1—Dilution as a show; A5—Diversity of Sizes; A2—Scientific principle; A7—Spherical egg, ovoid egg; A8—Aesthetic principle; A4—Economic principle; AH—Analogy and homology; CN—To eat and not be eaten; DC—Diversity of colors; DF—Diversity of shapes; DG—Genetic diversity versus uncertainty; ES—Speciation; G—Biodiversity that speaks portuguese; H—Entrance; SA—Artifical selection; SN—Natural selection; SS—Sexual selection; TS—Theatre of senses; TMA—By land, sea, and air. The odd-looking object in the center of the atrium is a complete whale skeleton suspended from the ceiling, spanning the ground and first floors.

The setting for the experiments was the first floor of the Hall. The floor has an area of 30 m × 30 m (900 m^2^), divided into 15 rooms (plus stairs and elevator) of various sizes and a central open area we call the atrium (c.f. Figure 2). The smallest rooms (SN and DC in Figure 2) measure 3.6 m × 7.3 m, while the largest (DF in Figure 2) spans 7.3 m × 12.5 m. The atrium is the main contributor to the total area, measuring 14.6 m× 14.6 m. Each of the rooms contains installations designed to provide innovative sensorial experiences while simultaneously conveying information on biodiversity and evolution. We installed beacons in 13 of these Regions of Interest (ROIs). The central atrium is the largest space and was further subdivided into 8 different ROIs, for a total of 21 ROIs. We used 27 out of 31 installed beacons (4 malfunctioned during the experiments) from three different manufacturers: Gimbal, Estimote, and Nordic, using either Google’s Eddystone Bluetooth or Apple’s iBeacon protocols. Figure 2 shows the floor layout and the Bluetooth beacons’ positions. The figure also shows the ROI labels used in the datasets to identify the corresponding areas on the floor.

**Figure 3:**
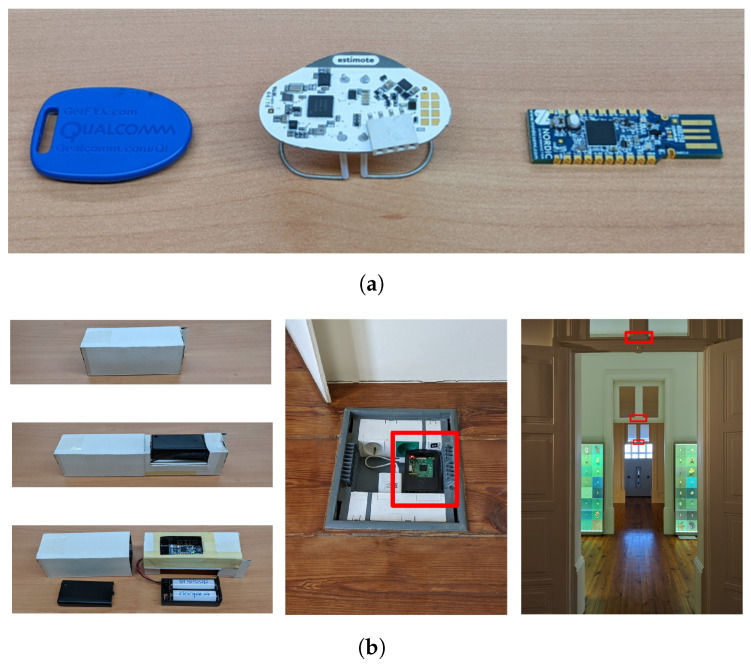
Beacons, capsules, and deployment. (**a**) Qualcomm, Estimote, and Nordic beacons. (**b**) A beacon capsule, the telemetry gateway, and beacon placement.

Figure 3 shows the beacons used (a) and deployment details (b). To improve the SNR of the RSSI data, we developed a partially shielded capsule to enclose the beacons so that the native isotropic signal was made more directional. These capsules had the additional goal of making the beacons inconspicuous. Figure 3b shows a Nordic beacon in a prototype capsule made from cardboard—a definitive version could use 3D-printed plastic—and the Raspberry Pi-based telemetry gateway and beacon capsules deployed over doors between rooms in the Hall.

**Figure 4:**
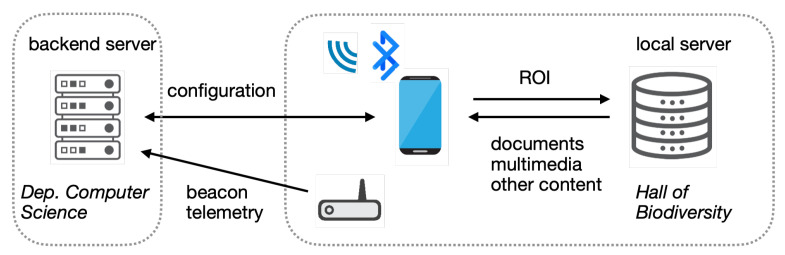
System architecture for the prototype ILBS installed at the Hall.

The deployment architecture (Figure 4) comprises a backend server (at our department) that hosts management software, including a web interface for administration and monitoring, and (at the Hall of Biodiversity) Bluetooth beacons, a telemetry gateway, mobile devices, and a local server that feeds extra content to the mobile devices based on their positions.

The physical deployment of the beacons presented some challenges due to the building’s nature; in addition to having restricted access to electrical outlets, the Hall building is classified as being of architectural and cultural interest. Therefore, beacons had to be installed so that they were nearly invisible. The rooms also have high ceilings, so that, on average, beacons were positioned in high places, e.g., over passages between rooms, resulting in lower RSSI values. On the positive side, this placement reduced interference from visitors and museum installations.

**Figure 5:**
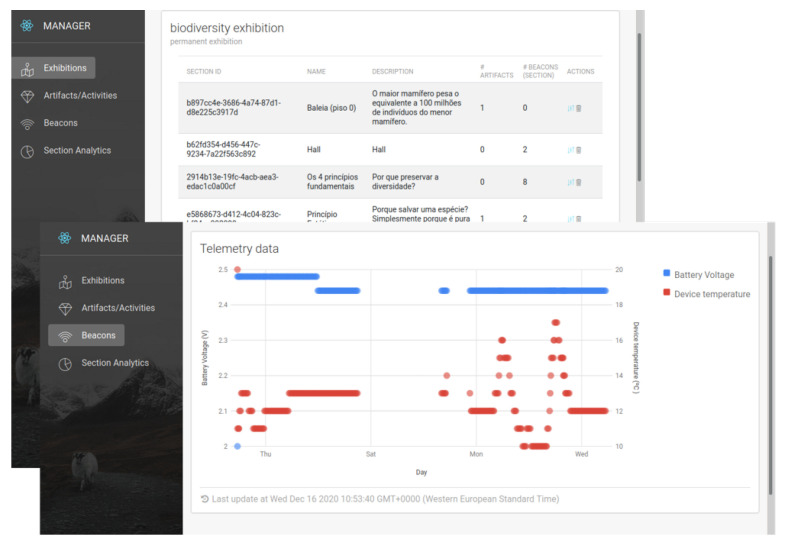
A snapshot of the ILBS’s administrative interface.

The backend server maintained a map of the beacon deployment in an internal database. The map associates beacons with specific ROIs in the Hall and allows for the seamless addition of new beacons or the removal of malfunctioning or redundant ones. Physically, the server was installed on a remote virtual machine with 4 GB of RAM and 2 CPUs running Linux, and was connected to the telemetry gateway located in the Hall building. From there, it received real-time data from the beacons (e.g., battery charge, microcontroller temperature), allowing an administrator to monitor deployment status and send commands to the beacons using a graphical interface, as depicted in Figure 5.

### 3.2. Datasets

We describe the data acquisition process and the construction of the datasets for BLE RSSI (hereafter BRSSI) and video data. Data collection was conducted in accordance with the museum’s guidelines and respecting the building’s historical and architectural classification. In addition to the beacon positioning restrictions already mentioned in Section 3.1, the lighting in each ROI was adapted for the installations therein and remained constant throughout the day; some ROIs had bright light, while others had a penumbral ambiance. It was not possible to collect data under varying lighting conditions. Moreover, data gathering could only be performed during periods when the Hall had no visitors to minimize disruption (typically after closing time).

#### 3.2.1. Data Acquisition

The data acquisition was performed using a custom smartphone app written in Kotlin and running on the Android operating system, developed with Android Studio. The app is capable of recording video frames and BRSSI measurements simultaneously. All BRSSI measurements were recorded (no sampling took place), while the video was captured at 30 frames per second. For each recording session, two files were written to disk: (1) an MP4 format file containing the recorded video frames, and (2) a CSV file containing BRSSI measurements over the same period with records of the form: [timestamp, beacon-id, brssi]. Using the two files after data acquisition, we cross-referenced video frames and BRSSI measurements within the same time frame, i.e., by aligning timestamped BRSSI measurements with the corresponding video frame.

**Figure 6:**
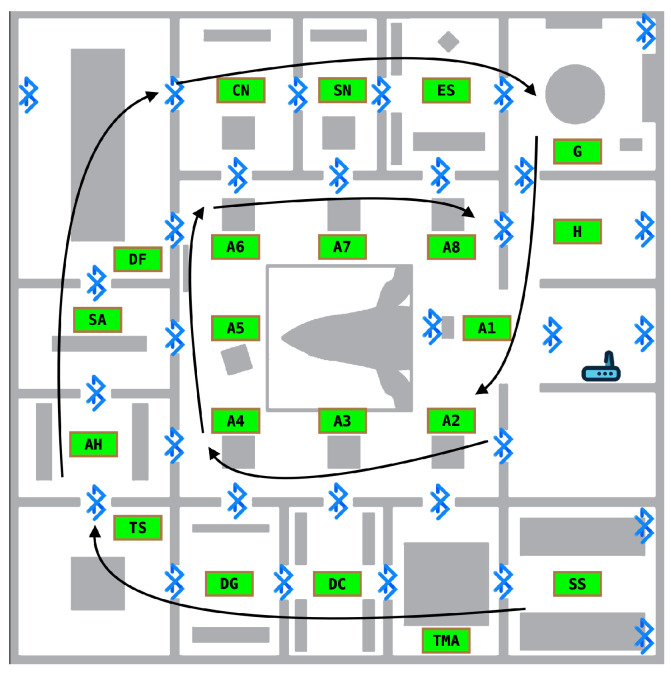
The ROI traversal pattern used for simulated visitor walks.

We performed two types of data acquisition using two smartphones: a Google Pixel 4 and a Xiaomi Redmi 9T. We first acquired data in individual ROIs for model training, consisting of circular movements around each ROI lasting approximately 2 min. The purpose was to define a base dataset used for training and a base test set. Every room and installation therein was carefully recorded on video. We also acquired data spanning all ROIs with a walking pattern simulating more realistic visits to the Hall. This spiral-like pattern is illustrated in Figure 6. First, the lateral rooms were traversed starting from SS and ending in H, followed by the atrium areas from A1 to A8. Unlike the base test dataset, there was no fixed pattern of movement within each ROI, and no attempt was made to record every installation on video. Two independent test sets were defined, one walk per device model.

#### 3.2.2. Dataset Construction

To build the datasets, we first conducted a pre-processing step on the acquired raw data. RSSI and video data were first annotated with the ground truth ROI labels, which are the required granularity for the ILBS. For the video data, we then picked one frame per second of recording. For the same 1 s time window and for each beacon detected, we averaged all corresponding BRSSI values. Beacons not detected during these 1-s periods were assigned a BRSSI default value of −200 dBm, a value well below the standard range of BRSSI measurements, typically between −30 and −120 dBm. Beyond this scheme of averaging RSSI values over 1-s periods and default value assignments for absent beacon signals, no other type of feature engineering was employed.

The resulting distilled data provided our training and test datasets. First, the data for individual ROI defined two different datasets, the base training set (TR) and the base test set (TS), with an 80–20% train–test split. As for the data from the walks, they were divided into two test sets according to the device used: Google Pixel (PW) and Xiaomi Redmi (RW).

The characteristics of these datasets are summarized in Table 1. For each dataset, the time span of the data acquisition is indicated, along with the number of data items. Note that the time span is the same for TR and TS, since both are splits of the same base dataset.

### 3.3. Models

Using the datasets mentioned above, we derived three types of models using BRSSI readings and/or video frames: (1) models trained only with BRSSI data, using the scikit-learn API [36] and standard classification approaches; (2) CNN models trained with video frames, using the TensorFlow API [37] and a CNN transfer learning approach, and (3) hybrid models that combined the outputs of BRSSI and CNN models using ensemble methods [38].

#### 3.3.1. BRSSI Models

For the BRSSI models, we considered several types of established classifier models and their corresponding core parameters available through the scikit-learn API. The models are listed in Table 2. The set of models considered includes some of the most common types for classification and regression tasks in the scikit-learn API. The various classifier types may capture different traits in the data, for details see [39]. For instance, tree-based ensembles (AdaBoost, Decision Trees, Gradient Boost, Random Forests) are good at modeling non-linear relationships and interactions, SVM-based methods (linear and RBF SVM) can handle high-dimensional state spaces but may have issues handling non-linear relationships, and the other models (kNN and multi-layer perceptrons) are well-suited to capture complex patterns. At this stage, we aim to identify which model types may be more adequate for the problem at stake without prior assumptions on or analysis of the data, seeking to establish baseline results. A grid-search is performed for each model type, using different values for the core parameters. The parameters are also listed in Table 2, and the values can be found in our supplementary material [10] (see the BRSSI_train.ipynb notebook). Grid-search exhaustively combines values for all search parameters, looking for the best possible model.

**Figure 7:**
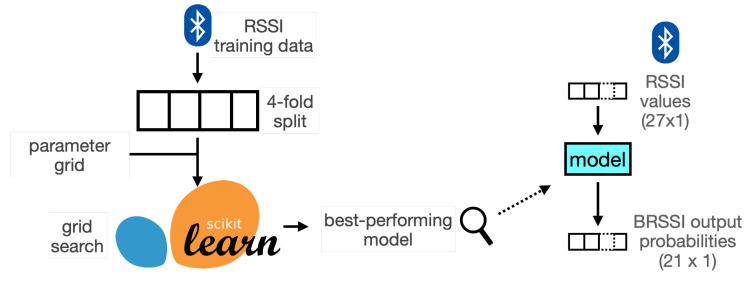
BRSSI models derived with scikit-learn.

The overall grid-search process is illustrated in Figure 7. For each parameter value combination, a 4-fold cross-validation strategy is used to measure accuracy, i.e., the data is partitioned into 4 equal splits, such that a different model was derived using 3 of the splits as proper training data and the remaining split was used as test data to evaluate accuracy (as illustrated also in the notebook). The average accuracy of the 4 splits is used as the overall score for the parameter combination at stake. For instance, in the case of Random Forest, we derived 24=4×2×3 different models accounting for 4 splits, 2 grid-search parameters, and 3 values for each grid-search parameter. The best-performing model for each classifier type is chosen using this grid-search process. Each derived model takes a vector of 27 BRSSI measurements (one from each beacon in the venue) and outputs a vector of 21 probabilities (one for each ROI in the venue).

#### 3.3.2. CNN Models

**Figure 8:**
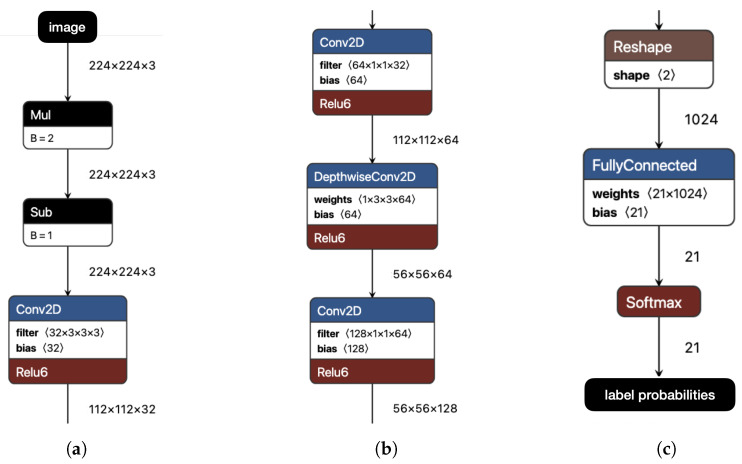
Layers of the MobileNetV1 CNN (fragments). (**a**) Initial (including input layer). (**b**) Intermediate. (**c**) Final (including output layer).

For models that process video frames, we employ convolutional neural networks (CNNs). CNNs are a widely used class of neural networks, particularly effective for image recognition and related tasks. As an illustrative example, consider fragments of the MobileNetV1 architecture [40] shown in Figure 8, one of the models we use, as described further below. The figure displays three fragments of the CNN: the input/initial layers (a), intermediate layers (b), and final/output layers (c). Each box represents a neuron type within the CNN, functioning as a mapping from an input tensor (a multi-dimensional array) to an output tensor. These functions are parameterized by internal weights, which are iteratively optimized through backpropagation during training. A defining feature of CNNs is their use of convolutional functions, which are very effective for capturing image patterns such as edges. In the simplest case—convolutions over 2D matrices—each element of the output (the feature map) is computed as the dot product between a sliding input window and a filter determined by the neuron’s weights. In later stages, CNNs typically produce a compact feature vector, which is passed to the final output layer through fully connected weights, as shown in (c). The output layer is an array of probabilities assigned to each label of the training domain (one value per label). For further details, see Chapter 9 of [41].

**Figure 9:**
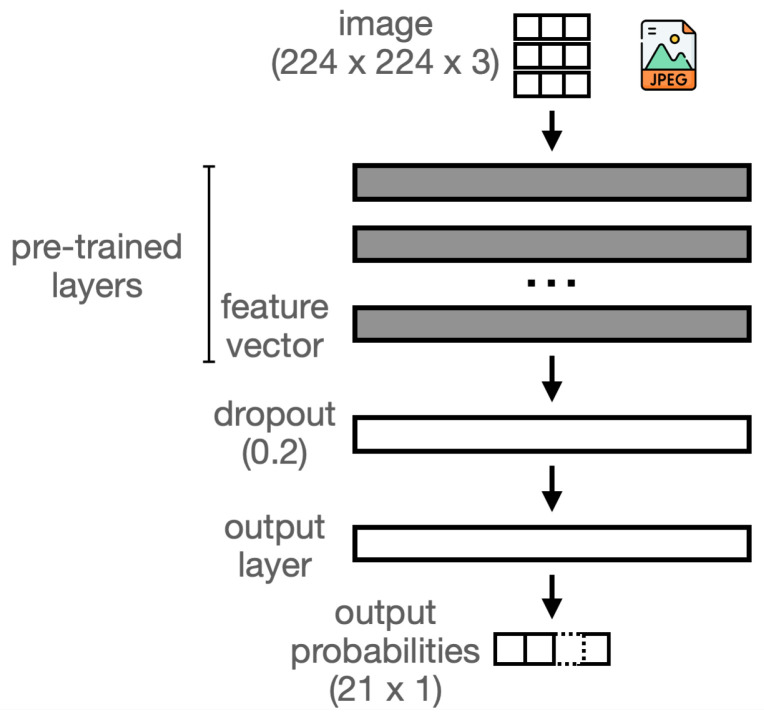
CNN models trained using transfer learning.

For the derivation of CNN models, we resort to transfer learning instead of the standard CNN training process from scratch. In this approach, a pre-trained CNN is reused in a different domain by replacing only the final output layer with a new one specific to that domain. This is illustrated in Figure 9. If the CNN has been pre-trained on a large and general dataset, it will capture generic features encoded in a feature vector, a level above the output layer. The feature vector often captures generic representations that can adapt to new domains. Only the weights of the fully connected interconnection between the feature vector layer and the domain-specific output layer need training, resulting in a small computation time; all other layers and corresponding interconnection weights are frozen. In a variation known as fine-tuning, which we did not employ, the remaining CNN layers can also be adjusted.

For our models, we consider transfer learning based on trained instances of some of the most popular state-of-the-art CNN architectures, listed in Table 3. For homogeneity, all CNNs obey the following conditions: all were obtained from Google’s Kaggle repository [42]; all were pre-trained on the ImageNet ILSVRC-2012 dataset [43]; and all have a 224×224×3 input shape dimension. Images are resized to a size of 224×224 and the third dimension relates to the Red–Green–Blue RGB color values for each image pixel. As shown in Table 3, these CNNs differ in their internal architecture (e.g., depth, layer types) and in the size of their feature vectors (i.e., number of features captured by the model). The transfer learning process was programmed using the TensorFlow Keras API, as defined in the CNN_Train.ipynb notebook of the supplementary material [10]. We follow a standard programming recipe for transfer learning using the TensorFlow API (cf. [44]). Essentially, a pre-trained CNN is loaded without its output layer, and a new output layer is defined with a shape that accounts for the new domain at stake (there are 21 ROIs in our case), and (only) the weights between the feature vector and new output vector are trained (all others are frozen/reused). This basic strategy is refined in our case by a dropout layer between the feature vector and output layer to prevent overfitting, as also shown in Figure 9. This is a standard technique by which a fraction of the weights is randomly disabled during training. All CNNs were retrained for 25 epochs, and a dropout factor of 0.2 was used.

#### 3.3.3. Hybrid Models

**Figure 10:**
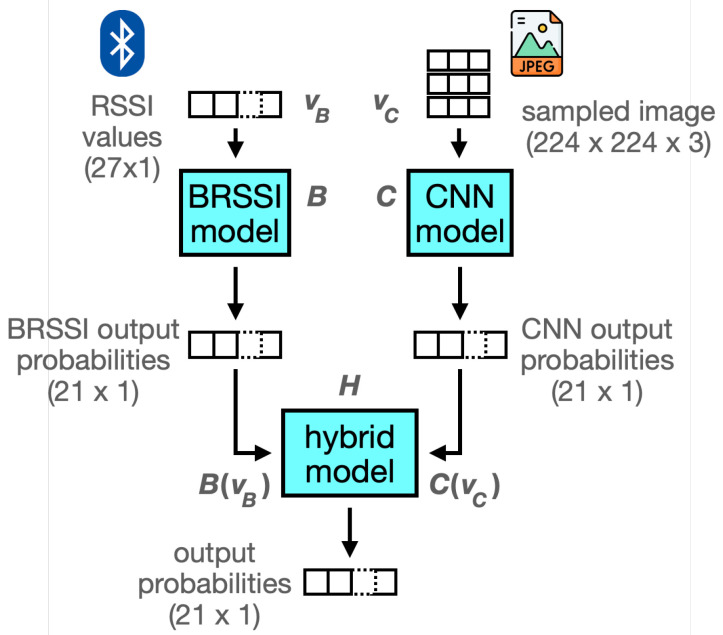
Synthesis of a hybrid model from the BRSSI and CNN models.

We consider hybrid models that receive BRSSI measurements and camera images using ensemble methods [38,45]. These combine the outputs of the BRSSI and CNN models, as illustrated in Figure 10. Specifically, for simultaneous BRSSI and image data, we first feed each type of data to one of the BRSSI models and one of the CNN models. The two outputs obtained, each a probability score for the museum room labels, are fed to the hybrid model to produce a new probability score. In summary, using the notation in the figure, given BRSSI values $v_{B}$ and image data $v_{C}$, the output of a hybrid model *H* is $H(B\left(v_{B}\right),C\left(v_{C}\right))$, where *B* and *C* are the BRSSI and CNN models, respectively. We considered two approaches to implement *H*: (1) soft voting, which works by simply averaging the outputs of the BRSSI and CNN models, i.e., the outputs are combined with equal weights of 0.5, and (2) stacking, which trains a meta-model using the outputs of the BRSSI and CNN models. For the latter approach, we considered meta-models using three approaches: logistic regression, which tends to be simple and efficient but may not capture non-linear relationships among the base model outputs; K-nearest neighbors, which may capture structural relationships among the base model predictions; and Random Forest, which may capture non-linear relationships between the base model predictions.

## 4. Results

In this section, we present the results obtained for the BRSSI, CNN, and hybrid models. The metric used for performance analysis is accuracy, defined as the fraction of correct predictions output by a model:

$$\mathrm{accuracy}=\frac{\mathrm{TP}+\mathrm{TN}}{\mathrm{TP}+\mathrm{TN}+\mathrm{FP}+\mathrm{FN}}$$

where TP, TN, FP, and FN denote true positives, true negatives, false positives, and false negatives, respectively. A prediction for a given input data is the label with the highest output score returned by the model, and is correct if it matches the ground truth associated with that data item. Beyond these baseline results, we also analyze the behavior of the models as a function of the ROIs and the devices used for capturing data. The former analyzes the sensitivity of BRSSI models in the presence of multipath signals and Bluetooth coverage, and of CNN models in open areas, where multiple installations may be visible and induce confusion. The latter highlights the impact of device-specific technology, such as transducers and photographic sensors, on the performance of the models. Finally, we also report on the models’ footprint in terms of disk storage and prediction latency, providing insight into their computational requirements for future use in concrete application deployments.

### 4.1. BRSSI Model Results

**Figure 11:**
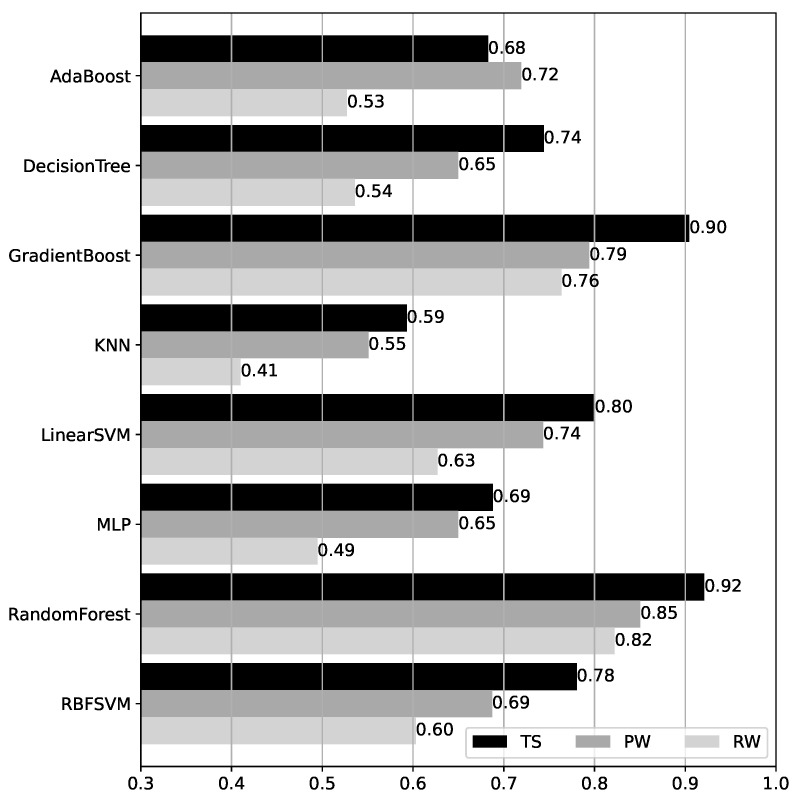
Accuracy of the BRSSI models over the test datasets.

Accuracy results for the BRSSI models over the test datasets are shown in Figure 11. We first observe that, except for AdaBoost, the accuracy for TS is consistently higher than for the RW and PW walk datasets. This is unsurprising since TS data corresponds to data acquired under the same conditions as the model training data, which is not the case for PW and RW (cf. Section 3.2). Moreover, comparing PW and RW, the accuracy for PW is always higher, with a very significant difference except in the case of the GradientBoost and RandomForest models, where this difference is only of 0.03 (0.79 vs. 0.76 for GradientBoost, and 0.85 vs. 0.82 for RandomForest). These two models also yield the best overall performance. Their accuracy values exceed 0.75 in all cases and reach 0.90 or higher for TS, with RandomForest outperforming GradientBoost slightly in all datasets. The remaining models tend to have significantly lower accuracy, ranging from 0.59 to 0.80 for TS, 0.55 to 0.74 for PW, and 0.41 to 0.60 for RW.

### 4.2. CNN Model Results

**Figure 12:**
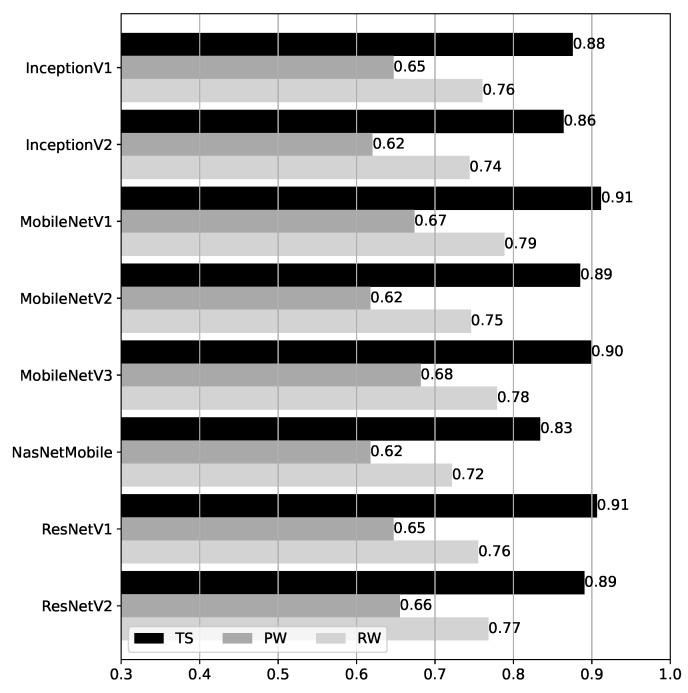
Accuracy of the CNN models over the test datasets.

Accuracy results for the CNN models over the test datasets are shown in Figure 12. Compared to the BRSSI models, the results for the CNN models are more homogeneous, especially considering each dataset individually. Despite the different CNN network architectures, the underlying abstractions and model capabilities are similar (cf. Section 3.3.2). The overall accuracy ranges from 0.83 to 0.91 for TS, 0.62 to 0.68 for PW, and 0.72 to 0.79 for RW. The best-performing models are MobileNetV1 and MobileNetV3, but only by a small margin. For all models, the accuracy is highest for TS, as expected and consistent with the BRSSI models’ results. Interestingly, all models performed better for RW than for PW. This behavior is exactly the opposite of that of the BRSSI models.

### 4.3. Hybrid Model Results

**Figure 13:**
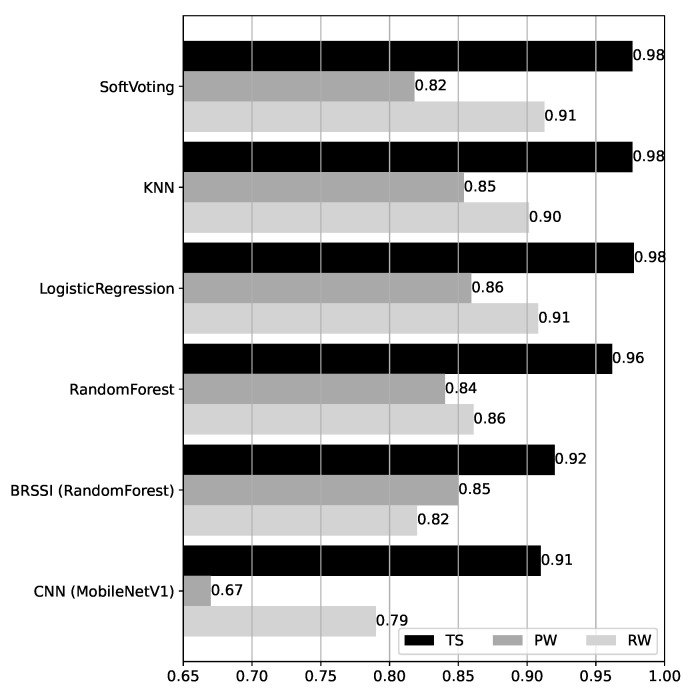
Accuracy of the hybrid models over the test datasets.

Accuracy results for the hybrid models over the test datasets are shown in Figure 13. The hybrid models are defined using ensemble methods (cf. Section 3.3.2), combining the RandomForest BRSSI model and the MobileNetV1 CNN model, the best-performing models for BRSSI and image data, respectively. The accuracy results for these baseline models are repeated at the bottom of the figure for easy comparison. First, we observe that the results are quite homogeneous across the hybrid models, with differences in accuracy across datasets not exceeding 0.05 (the largest being 0.91 vs. 0.86 for RW). Moreover, the accuracy is at least 0.96 for TS, 0.82 for PW, and 0.86 for RW. Compared to the baseline BRSSI and CNN models, their hybrid counterparts show clear improvements, except in the case of PW/BRSSI (baseline accuracy of 0.85), where the accuracy is slightly worse for RandomForest (0.84) and for SoftVoting (0.82).

**Figure 14:**
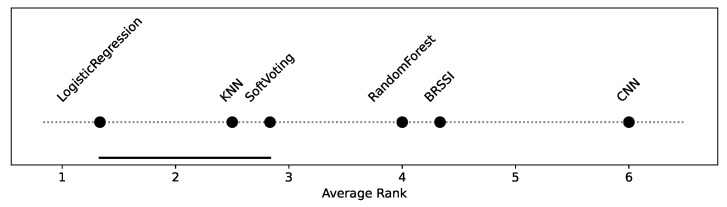
Critical difference diagram assessing the statistical significance of hybrid models.

To assess the improvement introduced by the hybrid models, we consider a statistical significance analysis. The analysis code is provided in the HM_SSAnalysis.ipynb of the supplementary material [10]. First, a Friedman omnibus test is performed, considering the accuracies of all models over all test datasets in Figure 13. The Friedman test yields a p-value lower than 0.5 (p=0.041), indicating there is a statistically significant difference among models. We then conducted post hoc pairwise Wilcoxon tests and derived the corresponding critical difference diagram shown in Figure 14. As shown in the diagram, there is no statistically significant difference between the LogisticRegression, SoftVoting, and kNN models (they form the cluster indicated by the bottom line connecting them). Otherwise, there are statistically significant differences between models, i.e., between the clustered models and all others, and all others pairwise. In particular, and this is a core aspect, the improvements observed for the hybrid models relative to the singleton BRSSI and CNN models are statistically significant.

### 4.4. Complementary Results

#### 4.4.1. Model Accuracy vs. Device Type

**Figure 15:**
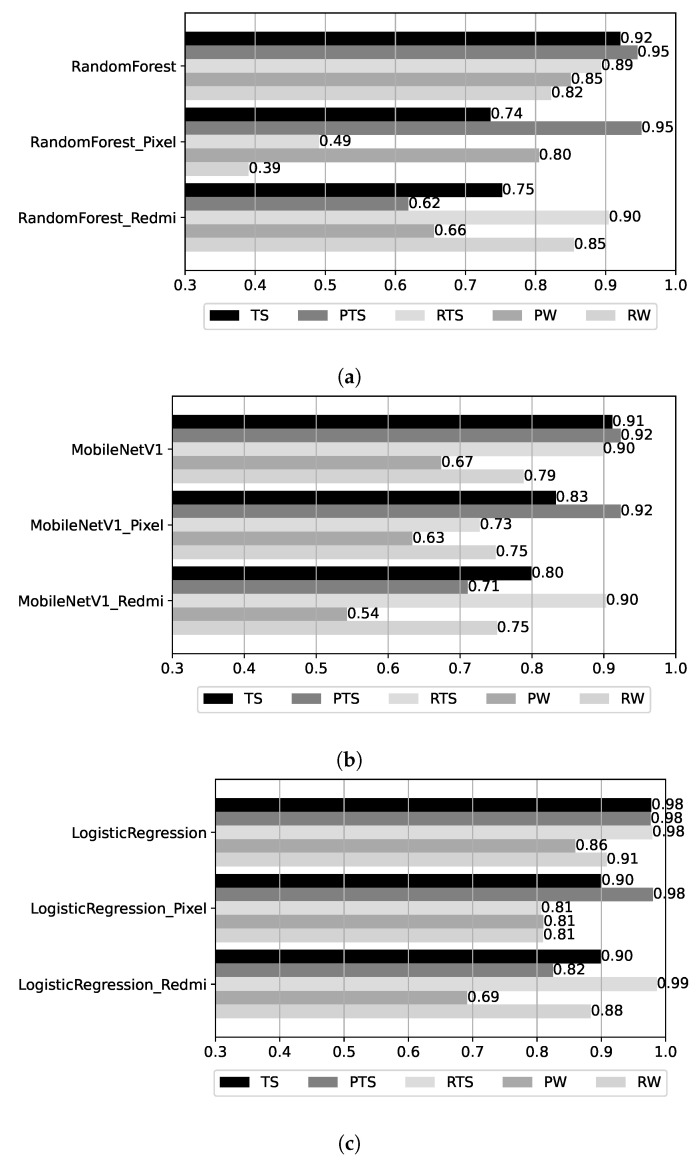
Results as a function of device and test datasets. (**a**) BRSSI models. (**b**) CNN models. (**c**) Hybrid models.

During the analysis above, we observed significant differences between the results obtained using data from the two smartphones at hand: the Google Pixel and Xiaomi Redmi. To study this effect, we derived models using data from each device individually. For these models, we measured accuracy over the partitions of the base test dataset that pertain to each device (PTS and RTS), as well as for the walk datasets that are already device-specific (PW and RW). The derived BRSSI, CNN, and hybrid models are instances of the best-performing variants reported in previous sections: RandomForest for BRSSI, MobileNetV1 for CNN, and LogisticRegression for hybrid. Figure 15 shows the results for the three test datasets, as in previous sections, but also for the Pixel TS (PTS) and Redmi TS (RTS) partitions of the base test set (TS). In each plot, the accuracy of three models for all datasets is shown: (top) for the model created with both Pixel and Redmi training data; (middle) a model created using Pixel data only; and (bottom) a model created using Redmi data only.

Observing the results for PTS and RTS, the models trained only with Pixel data have much better accuracy for PTS (the Pixel partition of TS) than for RTS: 0.95 vs. 0.49 for the BRSSI model (RandomForest_Pixel), 0.92 vs. 0.73 for the CNN model (MobileNetV1_Pixel), and 0.98 vs. 0.81 for the hybrid model (LogisticRegression_Pixel). A similar situation is observed for models trained only with Redmi data, where the accuracy is much better for RTS than PTS: 0.90 vs. 0.62 for the BRSSI model (RandomForest_Redmi), 0.90 vs. 0.71 for the CNN model (MobileNetV1_Redmi), and 0.99 vs. 0.82 for the hybrid model (LogisticRegression_Redmi).

**Figure 16:**
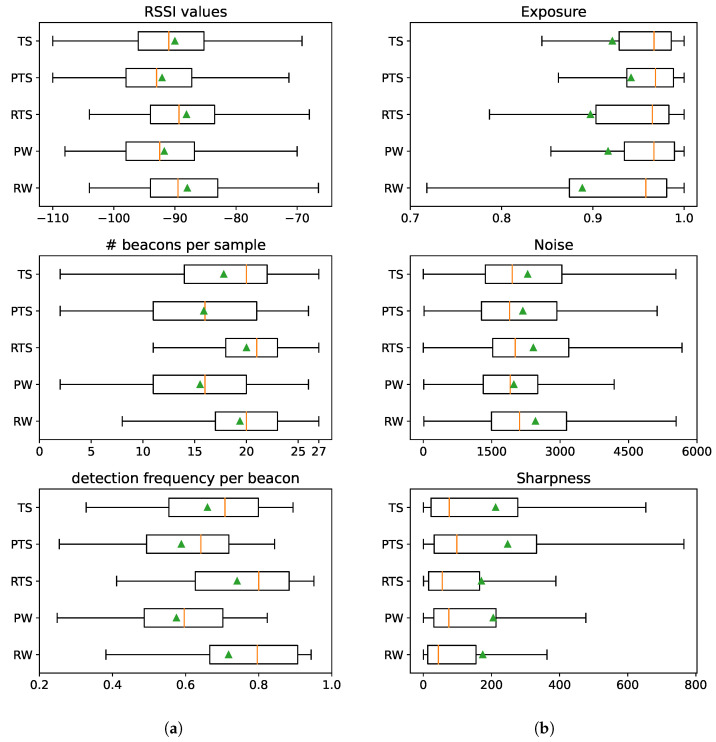
Test data quality indicators as a function of the test dataset. (**a**) BRSSI data. (**b**) Image data.

The observed patterns in the results for the Pixel and Redmi datasets likely stem from differences in the hardware components, namely, the radios and imaging sensors. To analyze if this is the case, we conducted an analysis of RSSI and image data, with results shown in Figure 16; the corresponding code can be found in the BRSSI_DataAnalysis.ipynb and CNN_DataAnalysis.ipynb notebooks of the supplementary material [10].

The box plots in Figure 16a (left) examine RSSI data samples in terms of the distributions of RSSI signal received (top), number of beacons detected per data sample (middle), and the frequency of individual beacon detection (for each of the 27 beacons) in samples (bottom). We observe that the Pixel device (PTS and PW datasets) performs worse than the Redmi device (RTS and RW datasets) when detecting Bluetooth beacons. The detected RSSI signal strength tends to be lower, whereas the number of beacons detected per sample and the frequency per individual beacon are visibly lower. The distinct data patterns explain why BRSSI models created using only Pixel or Redmi data, as illustrated for the BRSSI models in Figure 15a, perform much worse on data originating from the other device.

Regarding the image data feeding the CNN models, Figure 16b (right) presents the distribution of values regarding image exposure (top), noise (middle), and sharpness (bottom). The results show that images acquired with the Pixel device (PTS and PW datasets) tend to have longer exposures, less noise, and better sharpness when compared to those from the Redmi device (RTS and RW datasets). The Pixel 4’s camera, unlike the Redmi’s, features built-in optical image stabilization, which enables longer exposures to be taken, especially with a moving device, resulting in crisper and cleaner (less noisy) images. CNN-based image classification algorithms are known to be sensitive to noise levels in the training and input images [46,47]. Thus, as in the BRSSI models, CNN models created using only Pixel or Redmi data will tend to perform worse over data originating from the other device, as illustrated in Figure 15b.

#### 4.4.2. Model Accuracy vs. Location in the Hall

**Figure 17:**
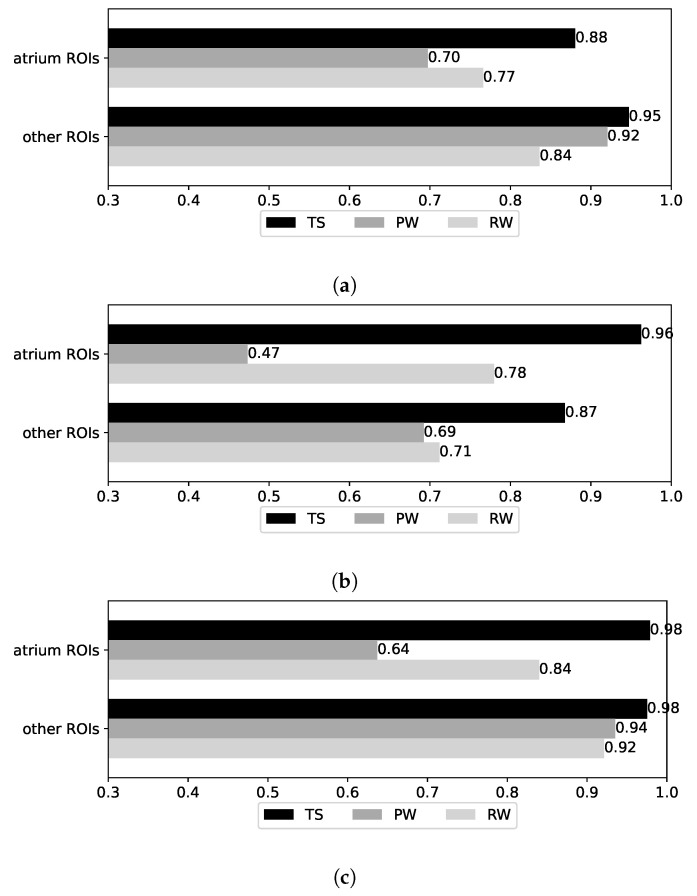
Results for atrium and non-atrium ROIs. (**a**) BRSSI (RandomForest). (**b**) CNN model (MobileNetV1). (**c**) Hybrid model (LogisticRegression).

We now measure the accuracy of the models in the ROIs we defined. For each of the datasets in Figure 17, we consider two cases: the atrium ROIs vs. other (non-atrium) ROIs. Recall that (cf. Figure 2) the A1-A8 ROIs are co-located in a large atrium room, while the other ROIs are each located in their own rooms. In the absence of obstacles, the accuracy of the atrium ROIs may thus be affected by similarities in Bluetooth RSSI signals and background/foreground objects captured in video. Indeed, we find that the accuracy is lower in the atrium ROIs for all datasets in the BRSSI and hybrid models (Figure 17a and Figure 17c, respectively). As for the CNN model (Figure 17b), the results are less clear-cut: the accuracy is higher for the atrium labels for the TS and RW datasets, and lower only in the case of the PW dataset.

**Figure 18:**
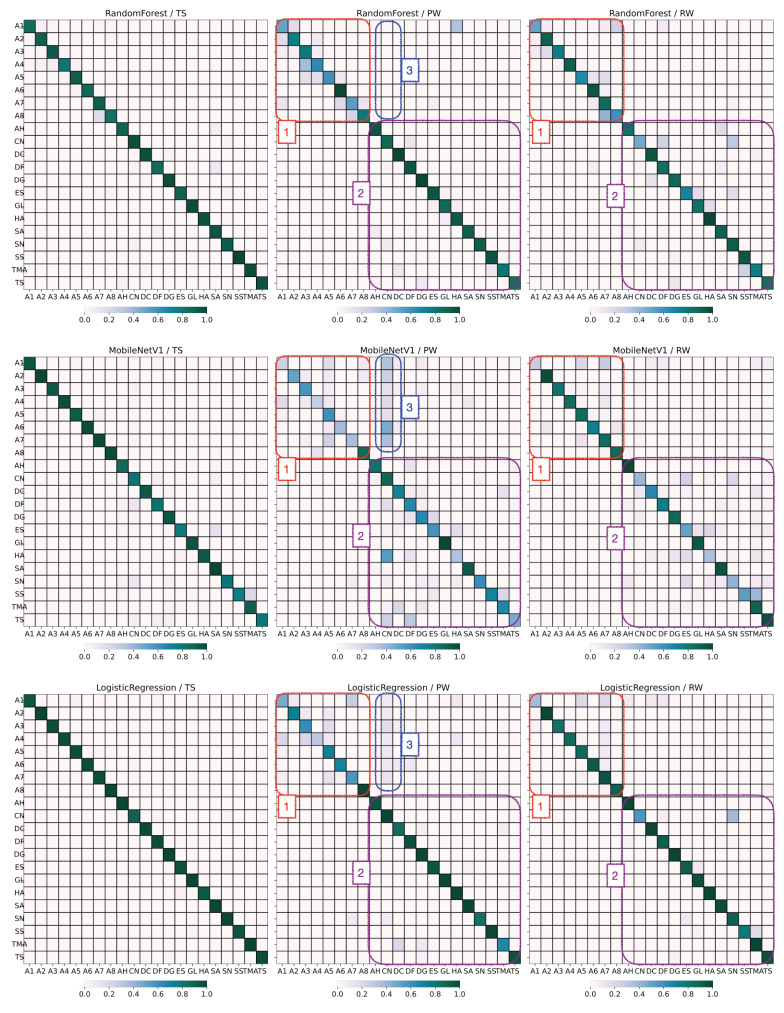
Confusion matrices for model/test-dataset combinations. (**Top row**): BRSSI model, (**middle row**): video model, and (**bottom row**): hybrid model. Boxes: **(1)** atrium labels, **(2)** non-atrium labels, and **(3)** CN ROI.

Figure 18 shows the confusion matrices for each model-dataset combination. For each matrix, the x-axis indicates the predicted ROI, the y-axis indicates the ground truth ROI, and the diagonal square for each ROI indicates the fraction of correctly classified items. For the TS dataset (confusion matrices shown in the left column), the performance is relatively uniform with only slight dispersion from the diagonal.

For the PW dataset (middle column), we observe significant confusion in the atrium labels (box #1). For the BRSSI model (top row, middle matrix), the confusion in the atrium mostly stems from cells adjacent to the diagonal. These cells correspond to installations that are in the immediate vicinity of the ground truth. We attribute this to the difficulty of the BRSSI model in pinpointing the installation at stake, as its neighbors are very close by. In the case of the video model (middle row, middle matrix), this effect is also seen; however, confusion also extends to other installations in the atrium. Because the atrium is an open area, images of installations feature elements in the foreground or background that the model can identify and sometimes confuse with the ground truth. This confusion is partly inherited by the hybrid model, which performs slightly worse than the BRSSI model.

For the RW dataset (right column), while the atrium (box #1) remains problematic, but to a lesser degree than for PW, there is still some dispersion due to other ROIs (e.g., CN, ES, SS, TMA—box #2). This dispersion is also visible for the PW dataset using the video model. For RW, the hybrid model nicely handles these cases and significantly improves accuracy. The lower performance of the models for the PW and RW datasets compared to TS was expected, considering the way the datasets were produced, as explained in Section 3.2.1.

Finally, there is some confusion with respect to the ROI CN in the PW test set, as it is misclassified in images from the atrium (box #3). We checked the video frames for PW and RW to investigate this anomaly and noticed that, in PW, the door connecting CN to the atrium was open. Objects from that room were visible in those frames, which caused the CNN model to misclassify them. This effect was then inherited, but diminished, by the hybrid model. The anomaly is not detected for RW.

#### 4.4.3. Storage and Latency Footprint

Table 4 presents data on the computational footprint for the RandomForest BRSSI, MobileNetV1 CNN, and hybrid LogisticRegression models. It lists the disk storage required by the models in MB, and the prediction latency in milliseconds (ms) per input sample evaluated over the TS dataset on a Linux machine with two cores and 8 GB of RAM; these characteristics are an approximation of the characteristics of low-end smartphones found in the market. In terms of disk space, the RandomForest and MobileNetV1 models have a non-negligible size, but one that is still perfectly manageable for modern embedded devices and smartphones (less than 100 MB). Regarding prediction latency, while the values for RandomForest and LogisticRegression are negligible (<1 ms), the MobileNetV1 model has a latency of 493 ms. This translates to a data processing frequency of approximately two images per second, which we consider adequate for future deployment of the models in a concrete museum setting.

## 5. Conclusions

Using RSSI and video frame datasets, we generated multiple models for an IPS at the Hall of Biodiversity, a unit of the Museum of Natural History and Science of the University of Porto. The RSSI data originated from a deployment of Bluetooth beacons on one of the building’s floors. The video data were obtained using the cameras on mobile phones in the same locations. Both were collected and timestamped using a custom Android application. This raw data, after refinement and further processing, resulted in several training and test datasets that were used to generate the ML models with the help of scikit-learn and TensorFlow. We then tested the models to determine their predictive power and properties. The video and the best-performing RSSI models were then combined into an ensemble-based hybrid model using different fusion strategies. The RSSI dataset, the video dataset, and the Jupyter notebooks used in training and evaluating the models are available from a public repository registered at Zenodo [10].

All models provide high accuracy, typically above 0.9 for TS, the base test set, and 0.6–0.9 for PW and RW, the complementary test sets that simulate user walks and are therefore noisier. We observe clear differences in model accuracy originating from the Bluetooth and video data collected by the two devices. Our analysis reveals that this is due to variations in the hardware components, specifically the radios and cameras, resulting in differing patterns in the data used to derive models. Moreover, the results confirm the atrium as the most problematic space in the Hall for both the RSSI and video models. To a lesser degree, other ROIs were identified where one of the models struggles to produce an accurate prediction due to factors such as multipath propagation and insufficient lighting. Overall, we observe that the hybrid models consistently show statistically significant improvements in accuracy compared to the RSSI and CNN models. This highlights the benefits of multimodal data integration solutions in venues where there are major restrictions on deploying a beacon infrastructure and controlling environmental conditions such as lighting.

For this work, we were only allowed to gather RSSI and video information when no visitors were present in the Hall. One important research question concerns the behavior of the models when the Hall has visitors. Intuitively, their presence will impact radio-signal propagation and the quality of video taken on the premises. We aim to quantify the effect of such crowded environments on the accuracy of the models as a function of the number of visitors and their spatial distribution within the Hall.

Furthermore, we intend to experiment with more advanced state-of-the-art algorithms, especially using multimodal data. Another concern is to improve the granularity of the output, so that instead of obtaining the name of the room, we can obtain a definite Cartesian position inside the Hall. This might be interesting for situations where multiple installations are located in the same ROI, and extra spatial resolution is required. For this, alternative technologies to Bluetooth, such as Wi-Fi RTT or UWB, might be required. To mitigate the problems arising from heterogeneous hardware configurations of mobile devices, our methodology can be improved with techniques for data normalization and the use of more devices as sources of training data. Finally, we consider the use of these models in a future museum smartphone app. For this purpose, our results demonstrate that the models require modest storage space and exhibit low latencies even in devices with limited computational capabilities. An app deployment, however, raises other concerns such as user experience and security.

## Figures and Tables

**Table 1:** Datasets considered for model training and testing.

Id	Description	Time–Span (min:s)	#Items
TR	Base training data	94:12	4710
TS	Base test data	94:12	942
PW	Walk data (from Google Pixel)	6:04	364
RW	Walk data (from Xiaomi Redmi)	7:22	445

**Table 2:** BRSSI model types and parameters considered during grid-search.

Model	Grid-Search Parameters
AdaBoost	number of estimators (n_estimators), learning rate (learning_rate)
Decision Tree	max. features for node split consideration (max_features), min. samples for node split (min_samples_split)
Gradient Boost	number of estimators (n_estimators), learning rate (learning_rate)
K-Nearest Neighbors (kNN)	number of neighbors (n_neighbors), weight function used in prediction (weights), algorithm used to compute the nearest neighbors (algorithm).
Linear SVM	regularization parameter (C), max. iterations (max_iter)
Multi-layer Perceptron (MLP)	number of layers and per-layer configuration (hidden_layer_sizes), L2 regularization term (alpha)
Random Forest	number of estimators (n_estimators), max. tree depth (max_depth)
Radial Basis Function (RBF) SVM	regularization parameter (C), max. iterations (max_iter)

**Table 3:** Architectures considered for CNN models using transfer learning (T: number of tensors; FV: feature vector dimension; P: total CNN parameters, including pre-trained parameters, in millions).

Model	T	FV	P
InceptionV1	86	1024	5.6
InceptionV2	100	1024	10.1
MobileNetV1	34	1024	3.2
MobileNetV2	69	1280	2.3
MobileNetV3	113	1024	1.5
NasNet Mobile	568	1056	4.3
ResNetV1	84	2048	23.5
ResNetV2	118	2048	23.6

**Table 4:** Storage and latency footprint.

Model	Storage (MB)	Latency (ms)
RandomForest (BRSSI)	51.1	<1
MobileNetV1 (CNN)	13.8	493
LogisticRegression (hybrid)	<1	<1

## Data Availability

Our paper is supplemented by datasets and Jupyter notebooks, available from Zenodo. The URL is https://doi.org/10.5281/zenodo.15980462 (accessed on 18 July 2025).
